# Mutagenicity, genotoxicity and oxidative stress induced by pesticide industry wastewater using bacterial and plant bioassays

**DOI:** 10.1016/j.btre.2019.e00389

**Published:** 2019-10-24

**Authors:** Mohammad Tarique Zeyad, Murugan Kumar, Abdul Malik

**Affiliations:** a Department of Agricultural Microbiology, Faculty of Agricultural Sciences, Aligarh Muslim University, Aligarh, UP-202002, India; b National Bureau of Agriculturally Important Microorganisms, Kushmaur, Mau, UP-275103, India

**Keywords:** Genotoxicity, Oxidative stress, Gas chromatography, Plasmid nicking, CLSM, Pesticide wastewater

## Abstract

Atomic absorption spectrophotometer and gas chromatography analysis revealed the presence of heavy metals, organochlorine and organophosphate pesticides in industrial wastewater. XAD, Dichloromethane and n-Hexane extracted wastewater were analysed for genotoxic potential using Ames *Salmonella*/mammalian microsome test. The XAD concentrated sample displayed remarkable mutagenic activity compared to solvent assisted liquid–liquid extraction. Strain TA98 was found utmost sensitive towards all extracts. Wastewater induced chromosomal aberrations in roots of *Allium cepa* showed significant (*p* < 0.05) decrease in mitotic index. Seeds of *Vigna radiata* germinated on soft agar plates treated with different concentration of wastewater showed significant reduction in germination (52 %), seedling vigor index (76 %), radicle length (56 %), plumule length (47 %), biomass of radicle (64 %) and plumule (57%) at highest wastewater concentration. Propidium iodide stained *V. radiata* roots showed oxidative stress induced by wastewater under CLS microscopy. Further, genotoxicity of wastewater was confirmed by plasmid nicking assay using pBR322 plasmid.

## Highlights

• Genotoxicity assessment of wastewater using bacterial and plant bioassays was carried out.
• *Salmonella typhimurium* and plant models (*Allium cepa* and *Vigna radiata* roots systems) revealed the toxicity of wastewater.
• The findings corroborate the significant morpho, cyto and genotoxic potential of wastewater.
• Dose dependent increase in toxicity and mutagenicity was observed with wastewater.

## Graphical abstract

**Figure:**
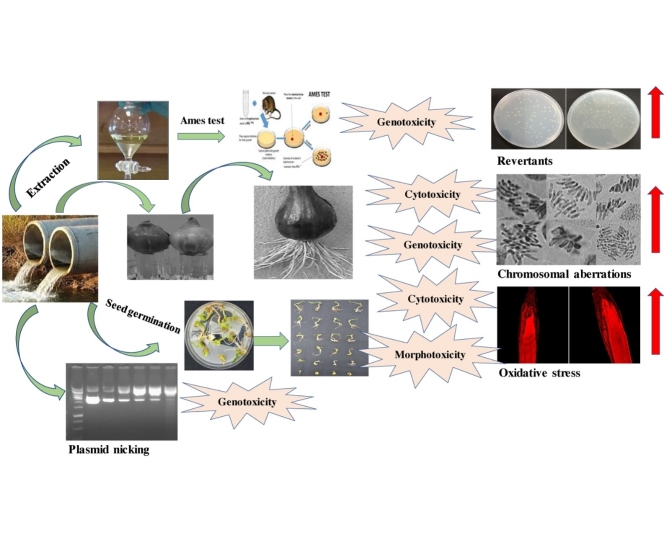

## 1 Introduction

One of the leading causes of water pollution is the unchecked release of wastewater from various industries into water bodies and many other environments [1,2]. The generation of wastewater is mostly due to rapidly growing industrial sector [3] for the development and expansion of the nation’s economy. Amongst the innumerable industries, the pesticide industry is counted as one of the key contributors of water contamination.

Organochlorine (OC) and organophosphorus (OP) pesticides are most important contaminants released by pesticide industry around the world as well as in India [4,5]. The existence of pesticide residues in water and soils impact on the vegetables as well as fruits and thus poses grave danger to human health. Many findings displayed that even very low level of pesticides cause natal defects [6]. Numerous scientific endeavours in the area of genotoxicity of wastewater suggested direct association with mutagenicity of pollutants into water bodies [7,8]. Several industrial wastewater effluents and sludges has shown high mutagenic potential [9,10].

With the fast pace in the development and era of modern mechanization the problem of pollution, specifically water pollution has been increased alarmingly in numerous developing countries including India [[11], [12], [13]]. A lot of toxicants in the environment act by damaging of DNA and therefore causing mutations [[14], [15], [16]]. Genotoxicity evaluation of industrial effluents on surface water indicates the presence of mixtures comprise of various toxic substances that may stance risk of hazard and carcinogenicity [17,18].

Biological assays with prokaryotic system detect mutagenic agents that persuade the gene level mutation and primarily damages the DNA. In contrast, eukaryotic based bioassay revealed exposure of a more degree of injury/impairment, variable from gene mutations to chromosomal aberrations and aneuploidies [19]. Applying both the prokaryotic and eukaryotic based detection systems reinforce and relate the observations to make certain if the substances actually hold any adverse effects on the genetic materials.

Ames *Salmonella*/microsomal test is extensively applied in examining the mutagenic potential of toxic chemicals [20,21]. *A. cepa* plant model is also extensively used for the evaluation of genotoxicity due to high sensitivity towards the xenobiotic compounds [22]. Mung bean (*Vigna radiata*) seed is another important short-term assay for genotoxicity evaluation using different parameters such as seed germination, seedling vigour index [7] to reflects the impacts of contaminants on the growth of plant. Many other *in-vitro* tests for the evaluation DNA damages are also routinely applied in the studies of wastewater monitoring. Among these tests, plasmid-nicking assay has been usually employed and delivers an effective indicator of genotoxicity [23]. By the means of both the prokaryotic and eukaryotic evaluation systems, it supports and corelate the observations and confirmed that the xenobiotic compounds/toxic chemicals severely affect the genes of both the systems.

Present study focused on the cytotoxicity, genotoxicity and phytotoxicity of pesticide industry wastewater collected from in the vicinity of Ghaziabad city, India, using different prokaryotic and eukaryotic assays. Additionally, plasmid nicking assay has also been used to evaluate the direct impact of wastewater on DNA integrity.

## 2 Materials and methods

### 2.1 Sample collection

Wastewater samples were collected from the open channels, receiving sewage from industrial area of Ghaziabad, situated about 10 km North of the Hindon River at latitude 28°40´ North and longitude 77°25´ East. A total 12 samples of wastewater were taken from January 2015 to June 2017 and transferred to the laboratory as described in standard methods [24]. Two litres of samples taken from five diverse points and make 10 L by composite mixing.

### 2.2 Physico-chemical characteristics of the wastewater

Physico-chemical properties of the wastewater such as total dissolve solid (TDS), pH, carbonate, bicarbonate, sulphate and chloride were carried out according to method adopted by Gupta [25].

### 2.3 Preparation of concentrated wastewater extracts

#### 2.3.1 XAD-concentration of wastewater

One litre of wastewater was used to concentrate organic constituents. Whatman filter paper no. 1 with pore size 11 μm and 0.45 μm pore size (Axiva, India) were used to filter the wastewater. The adsorbent columns were prepared by intermixing equal quantity of XAD-4 and XAD-8 [26]. Organic compounds present in the wastewater were adsorbed on the resins using methods described by Wilcox and Williamson [27]. The adsorbed organic compounds were eluted with 20 ml of acetone (HPLC grade) and then eluate was evaporated to dryness at room temperature (25 °C) under decreased pressure and further dissolved in Dimethyl sulfoxide (DMSO) (HPLC grade, SRL, India). The samples were filtered through membrane filter (0.22 μm) to sterilized and stored for further use at −20 °C.

#### 2.3.2 Liquid-liquid extraction

Industrial wastewater samples were sequentially extracted by means of different organic solvents such as Dichloromethane (DCM) and n-Hexane (HPLC grade, SRL, India) as defined in standard procedures [24]. Wastewater was vigorously shaken in a separating funnel with the solvent and was set aside to hold up till the aqueous (water) and organic solvent layers were separated. The solvent layer was collected in a 100 ml beaker then evaporate at 25 °C to concentrate up to 5 ml. The obtained extracts of wastewater were filter (pore size 0.22 μm) sterilized and stored at −20 °C for further use of genotoxicity testing [28].

### 2.4 Gas chromatographic (GC) analysis of pesticides in wastewater

Gas chromatography analysis of wastewater extracts was performed via GC-2010 gas chromatograph (Shimadzu, Japan). The parameter of the instrument and operating conditions are as follows: {column: Rtx-5MS, temperature: (injector: 290 °C, detector: 300 °C, oven: initial temperature 100 ◦C/min then increase 300 °C then 5 °C/ram, hold time: 1–9 min, carrier flow rate of gas helium: 21 ml/min, flow rate of carrier gas helium: 1.21 ml/min afterward makeup 30 ml/min)}. By comparing retention time of standards obtained from Sigma-Aldrich, peaks of samples were identified. Multi-standard of 20 organochlorine (CRM-47426) and nine organophosphorus (48,391) pesticide mixtures purchased from Sigma-Aldrich company containing Aldrin, α-BHC, β-BHC, σ-BHC, Endrin Aldehyde, Endrin, Endrin ketone, Chlordane, γ-Chlordane, Dieldrin, 4-4″ DDT, 4-4″ DDE, Lindane, Heptachlor, Heptachlor Epoxide, Endosulfan I, Endosulfan II, Endosulfan sulfate, Methoxychlor, (Organochlorine), Azinphos-methyl, Chlorpyriphos, Dichlorvos, Ethoprophos, Disulfoton, Parathion-methyl, Fenchlorphos, Prothiofos, Malathion (organophosphorus) and stored at 4◦C.

### 2.5 Ames/*Salmonella* mutagenicity test

The *Salmonella* mutagenicity test was performed as described by Maron and Ames [20] with minor changes as adopted earlier [29]. Five different doses of individual wastewater extract i.e., 2.5, 5, 10, 20 and 40 μL per plate (add 0.1 ml of the overnight grown bacterial culture) were incubated for 30 min at 37 °C in triplicate. Two ml of top agar with trace amount of biotin and histidine were added and poured onto plates of minimal glucose agar and incubated at 37 °C for 2–3 days. Positive control comprising bacterial culture and methyl methane sulfonate (MMS) and negative control includes bacteria and double distilled water. Parallel tests were also performed in the presence of S9 microsomal fraction to detect incidence of pro-mutagens in the samples containing 20 μL of S9 liver homogenate per plate.

### 2.6 *Allium cepa* anaphase-telophase test

To determine the toxic effect of wastewater, samples were tested using root tip cells of *A. cepa* as described by Fiskesjo [30]. The small bulbs of *A. cepa* (2n = 16) with 1.5–2.0 cm in diameter were purchased from local market. Before starting the assay, outer dead scales and dry bottom of *A. cepa* bulbs were detached without disturbing root primordia. The bulbs were put in beakers, comprising DD water. The basal portions of bulbs dipped into the water and allow to evolve for 2–3 days at room temperature (25 °C). The freshly grown roots upto 2 cm were used in this assay. The newly grown root tips were treated with several different concentration of wastewater i.e., 5, 10, 25, 50, and 100% for 3 days. Simultaneously, positive control with MMS and negative control with double distilled water were also performed in each test. After exposure to wastewater samples upto three days, root tips were selected randomly, fixed in the ratio of 3:1 ethanol and glacial acetic acid (v/v) and incubated for overnight at 4 °C. The fixed root tips were washed with tap water followed by heating for 2–3min in 1 N HCl then with DD water and stained with acetocarmine and observed under light microscope (Olympus, BX60). By observing approximately 6000 dividing cells (2000 cells per slide), mitotic index (MI) was calculated as follow:

$$\text{M}\text{i}\text{t}\text{o}\text{t}\text{i}\text{c}\;\text{i}\text{n}\text{d}\text{e}\text{x}\;(\text{\%})=\frac{\text{T}\text{o}\text{t}\text{a}\text{l}\;\text{N}\text{u}\text{m}\text{b}\text{e}\text{r}\;\text{o}\text{f}\;\text{D}\text{i}\text{v}\text{i}\text{d}\text{i}\text{n}\text{g}\;\text{C}\text{e}\text{l}\text{l}\text{s}}{\text{T}\text{o}\text{t}\text{a}\text{l}\;\text{N}\text{u}\text{m}\text{b}\text{e}\text{r}\;\text{o}\text{f}\;\text{C}\text{e}\text{l}\text{l}\text{s}\;\text{E}\text{x}\text{a}\text{m}\text{i}\text{n}\text{e}\text{d}}\times 100\;$$

Chromosomal aberrations were evaluated by observing approximately 300 dividing cells (preferably 100 cells per slide).

### 2.7 Phytotoxicity testing of wastewater

#### 2.7.1 Effect of wastewater on germination and growth of *Vigna radiata* (mung bean) under *in vitro* condition

Phytotoxicity of wastewater on seed germination was done according to the method of Kalyani et al [31]. Briefly, mung bean seeds (*Vigna radiata* L. Wilczek) were surface sterilized using 70% ethanol followed by 3% sodium hypochlorite solution for 3 min. The sterilized seeds were repeatedly washed through DD water. The sterilized seeds of mung bean were soaked in different concentrations of filtered wastewater for overnight and then placed on 0.7% agar plates. The agar plates were also prepared with sterile DD water (control). All the plates were incubated at room temperature (25 °C) with humidity upto 75%. After 5–7 days of incubation the emergence of seeds, length of plumule and radicle, and dry biomass were recorded.

$$\%\text{Germination}=\frac{\text{number of seeds germinated}}{\text{total number of seeds}}\times 100$$

The seedling vigor index (SVI) were calculated from percent germination of seeds [32].

SVI = (root length + shoot length) × % seed germination

#### 2.7.2 Oxidative damage induced by wastewater under confocal microscopy

In order to assess the oxidative damage induced by wastewater; confocal microscopy was used to observe the dead cells. Briefly, roots of *V. radiata* plants were grown on 0.7% agar plates amended with filtered wastewater of different concentration i.e., 10%. 25%, 50% and 100% for seven days [33]. Negative (DD water) and positive control (MMS) were also performed in each assay. After three time washing with phosphate buffer saline (PBS), propidium iodide (PI) were used to stain the root samples and fixed on a glass slide and observed under LSM-780 Confocal Microscope (Zeiss, Germany).

### 2.8 Plasmid nicking assay

The plasmid-nicking assay was performed as described by Siddiqui et al [23] with minor changes, the covalently closed circular pBR322 plasmid DNA (0.5 μg) was treated with different concentration of wastewater in a total volume of 25 μl for 3 h at room temperature. After incubation at room temperature, 5 μl of 5x tracking dye (40 mM EDTA, 0.05% bromophenol blue with glycerol 50% v/v) was mixed into the reaction tube and were run with one percent agarose on gel electrophoresis at 50 mA for 90 min followed by staining with ethidium bromide (0.5 μg/l). The DNA bands in the agarose gel were visualized on a BIO-RAD Chemi Doc XRS imaging system and photographed.

### 2.9 Statistical analysis

Mutagenic Index, induction factor (Mi) and mutagenic potential (*m*) were calculated as defined by Ansari and Malik [11].

$$\text{Mutagenic index}=\frac{\text{Number of his + revertants induced in the sample}}{\text{Number of his + revertants induced in the negative control}}$$

$$\text{Induction factor}\left(\text{Mi}\right)=\frac{\text{ln}\left(\text{n-c}\right)}{\text{c}}$$

Where “n” is no. of revertant bacterial colonies in the samples while “c” is the number of revertant colonies in control. The mutagenic potential of the wastewater samples was calculated as described by Khan et al [8].

The total number of *his^+^* revertant bacterial colonies in comparison to control was recognized by one-way analysis of variance (ANOVA) at p ≤ 0.05. Data were represented in terms of percent mitotic index and percentage of abnormal cells. In case of seed germination assay, percent germination of seed and plumule-radicle growth were represented as Mean ± Standard Deviation (SD) and analysed with Duncan Multiple Range Test (DMRT) were applied to analyse significance in the treatment sets as well as in contradiction of positive and negative control data.

## 3 Results

### 3.1 Physico-chemical and heavy metal analysis

The physico-chemical characteristics of wastewater is presented in the Table 1. Test samples displayed pH in the range of 7.0–7.3. The concentration of total dissolve solids, carbonate, bicarbonate, chloride and sulphate were recorded to be 767 mg L^−1^, 164.5 mg L^−1^, 70.47 mg L^−1^, 45.38 mg L^−1^ and 0.05 mg L^−1^, respectively. Atomic absorption spectrophotometric (AAS) analysis revealed the presence of numerous heavy metals i.e. Ni (0.45 mg L^−1^), Cu (0.13 mg L^−1^), Cr (1.91 mg L^−1^), Pb (1.17 mg L^−1^), Cd (0.02 mg L^−1^) and Zn (0.13 mg L^−1^), with concentration of Cd, Cr and Pb being pointedly higher than permissible limits as given by Unites States Environmental Protection Agency (US EPA).

**Table 1:** Physico-chemical and heavy metal analysis of industrial wastewater.

Parameters	Wastewater
pH	7.17 ± 0.1
TDS	767 ± 9.46
Carbonate	164.5 ± 6.41
Bicarbonate	70.47 ± 2.52
Chloride	45.38 ± 2.39
Sulphate	0.05 ± 0.001
Dissolve oxygen	2.27 ± 0.02
Free CO_2_	21.83 ± 0.62
Total CO_2_	26.24 ± 1.03
Nickle	0.45 ± 0.12
Cadmium	0.2 ± 0.01
Lead	1.17 ± 0.1
Copper	0.13 ± 0.08
Chromium	1.91 ± 0.3
Zinc	0.13 ± 0.01

All parameters except pH are in mg/L.

### 3.2 Determination of organochlorine and organophosphate pesticides

The gas chromatographic (GC) analysis revealed that the industrial wastewater contains several organochlorine pesticides such as α-BHC, β-BHC, σ-BHC, Aldrin, γ-Chlordane, Endosulfan I, Endosulfan II, Endosulfan sulfate, Dieldrin, Endrin Aldehyde, Endrin ketone at the concentration of 82.9, 38.01, 7.52, 108.6, 12.4, 4.37, 311.7, 22.49, 6.43, 125.8, 4.21 and 66.61, ng mL^−1^ respectively (Table 2), while organophosphorus pesticides Ethoprophos, Disulfoton, Parathion-methyl, Chlorpyrifos, Prothiofos were detected at the levels of 3.54, 22.4, 20.6, 8.93 and 321.5 (Table 2) ng mL^−1^, respectively. Many other unidentified peaks were also detected in the gas chromatograms of test samples showing the occurrence of more organic pollutants apart from pesticide.

**Table 2:** Concentration of pesticides in industrial wastewater as determined by gas chromatography.

Organochlorine (OC)	Concentration(ng/ml)	Organophosphate (OP)	Concentration(ng/ml)
α-BHC	82.9 ± 6.72	Dichlorvos	ND
β-BHC	38.01 ± 2.51	Ethoprophos	3.54 ± 0.67
Lindane	ND	Disulfoton	22.4 ± 1.83
σ-BHC	7.52 ± 0.73	Parathion-methyl	20.6 ± 2.01
Heptachlor	ND	Fenchlorphos	ND
Aldrin	108.6 ± 6.2	Chlorpyrifos	8.93 ± 1.55
Heptachlor epoxide	ND	Prothiofos	321.5 ± 33
γ-Chlordane	12.4 ± 2.1	Azinphos-methyl	ND
α-Chlordane	ND	Malathion	ND
Endosulfan I	4.37 ± 0.45		
4-4” DDE	ND		
Dieldrin	311.7 ± 49.7		
Endrin	ND		
Endosulfan II	22.49 ± 4.7		
4-4” DDD	ND		
Endrin aldehyde	6.43 ± 1.2		
Endosulfan sulfate	125.8 ± 22.3		
4-4” DDT	4.21 ± 0.57		
Endrin Ketone	66.61 ± 6.23		
Methoxychlor	ND		

ND = not detected.

### 3.3 Reversion of *Salmonella* tester strains

The wastewater samples were evaluated for their mutagenicity using *S. typhimurium* strains. Liquid-liquid extracted (DCM and n-hexane) and XAD concentrated samples were tested in the presence and absence of S9 fraction. In XAD concentrated samples, the number of revertant colonies increased upto 20 μL/plate with all the tester strains. The maximum number of revertants were observed in TA98 and showed mutagenic index of 13.11 (without S9 fraction) and 13.19 (with S9 fraction) among all the tester strains. The strain TA98 revealed highest response in terms of induction factor (2.50 without and with S9 fraction both) and mutagenic potential/slope (m) were observed 7.7 with S9 fraction and 6.8 without S9 fraction (Table 3). On the basis of induction factor and mutagenic index, the order of responsiveness both in the presence and absence of S9 fraction for XAD concentrated wastewater sample was as: TA98>TA97a>TA100>TA102>TA104. TA98 showed maximum responsiveness followed by TA100, TA97a, TA104 and TA102.

**Table 3:** Reversion of *Salmonella* tester strains in the presence of XAD concentrated wastewater.

Wastewater extract (μL/plate)											F value
Strain	S9	Control	2.5	5	10	20	40	Mi	m	LSD (*p*≤0.05)	
TA97a	–	88 ± 18	225 ± 27 (2.53)	297 ± 27 (3.35)	373 ± 18 (4.21)	456 ± 22 (5.14)	381 ± 12 (4.31)	1.43	5.87	29.8	281.6
	+	94 ± 11	241 ± 17 (2.56)	317 ± 17 (3.37)	405 ± 21 (4.31)	484 ± 24 (5.15)	407 ± 23 (4.33)	1.43	6.21	14.9	145.1
TA98	–	35 ± 6	221 ± 22 (6.26)	268 ± 25 (7.57)	367 ± 18 (10.37)	463 ± 14 (13.11)	382 ± 19 (10.41)	2.50	6.79	24.1	174.7
	+	39 ± 11	250 ± 15 (6.40)	299 ± 26 (7.65)	405 ± 22 (10.37)	515 ± 16 (13.19)	433 ± 20 (11.01)	2.50	7.71	29.6	121.5
TA100	–	127 ± 14	232 ± 18 (1.82)	301 ± 11 (2.37)	411 ± 19 (3.23)	501 ± 18 (3.94)	399 ± 27 (3.13)	1.08	5.92	27.5	85.6
	+	138 ± 16	253 ± 20 (1.83)	329 ± 22 (2.37)	451 ± 17 (3.26)	556 ± 14 (4.01)	453 ± 16 (3.27)	1.11	6.95	17.2	111.2
TA102	–	226 ± 12	324 ± 15 (1.43)	410 ± 21 (1.81)	497 ± 25 (2.20)	560 ± 19 (2.48)	432 ± 14 (1.91)	0.39	4.13	13.5	231.5
	+	241 ± 8	354 ± 17 (1.46)	441 ± 19 (1.83)	510 ± 24 (2.12)	598 ± 17 (2.48)	465 ± 17 (1.93)	0.39	4.47	10.8	118.9
TA104	–	301 ± 17	379 ± 19 (1.25)	464 ± 24 (1.54)	539 ± 21 (1.77)	625 ± 17 (2.07)	515 ± 22 (1.71)	0.07	4.68	19.5	165.4
	+	318 ± 16	408 ± 22 (1.28)	493 ± 21 (1.55)	568 ± 16 (1.78)	668 ± 24 (2.09)	569 ± 16 (1.78)	0.10	4.49	16.7	217.9

Values in parentheses are mutagenic index; Mi = induction factor; m = mutagenic potential; LSD = least significant difference.

The revertant colonies in the hexane extracted wastewater sample increased with increasing dose from 2.5 to 20 μL/plate, while at 40 μL/plate dose, revertant colonies decreased (Table 4). Mutagenic index in strain TA98 showed maximum response (12.09 without S9 and 12.15 with S9 fraction); induction factor (2.41 without S9 and 2.42 with S9 fraction); and mutagenic potential is 8.0 with S9 and 7.4 without S9 fraction (Table 4). The mutagenic index and induction factor, the response of tester strains showed dissimilar trends as observed in the XAD concentrated wastewater samples was as follows: TA98>TA97a>TA100>TA102>TA104.

**Table 4:** Reversion of *Salmonella* tester strains in the presence of hexane extracted wastewater.

Wastewater extract (μL/plate)											F value
Strain	S9	Control	2.5	5	10	20	40	Mi	m	LSD (*p*≤0.05)	
TA97a	–	88 ± 13	167 ± 19 (1.90)	204 ± 14 (2.32)	276 ± 13 (3.15)	347 ± 25 (3.96)	281 ± 12 (3.21)	1.07	4.2	36.2	214.1
	+	93 ± 11	191 ± 12 (2.06)	221 ± 15 (2.38)	293 ± 16 (3.16)	378 ± 22 (4.07)	323 ± 21 (3.48)	1.11	4.9	9.52	156.9
TA98	–	35 ± 7	144 ± 15 (4.08)	260 ± 23 (7.36)	344 ± 19 (9.73)	427 ± 15 (12.09)	384 ± 14 (10.85)	2.41	7.4	8.65	321.2
	+	38 ± 11	161 ± 9 (4.20)	286 ± 18 (7.47)	375 ± 13 (9.79)	466 ± 24 (12.15)	418 ± 20 (10.91)	2.42	8.0	12.8	286.5
TA100	–	130 ± 8	219 ± 10 (1.68)	302 ± 21 (2.31)	376 ± 18 (2.88)	443 ± 24 (3.40)	406 ± 23 (3.11)	0.88	5.9	7.69	123.2
	+	147 ± 12	253 ± 13 (1.73)	343 ± 21 (2.33)	424 ± 24 (2.89)	501 ± 17 (3.41)	458 ± 13 (3.12)	0.88	6.6	8.9	254.1
TA102	–	234 ± 9	291 ± 22 (1.24)	361 ± 18 (1.54)	431 ± 22 (1.83)	493 ± 17 (2.10)	433 ± 13 (1.84)	0.10	4.4	10.5	165.3
	+	246 ± 10	325 ± 15 (1.31)	396 ± 12 (1.60)	458 ± 22 (1.86)	532 ± 26 (2.15)	466 ± 19 (1.89)	0.15	4.7	11.9	154.8
TA104	–	311 ± 15	362 ± 18 (1.16)	413 ± 12 (1.32)	467 ± 21 (1.49)	530 ± 19 (1.70)	476 ± 19 (1.53)	−0.35	3.7	13.0	218.9
	+	332 ± 11	392 ± 20 (1.17)	456 ± 25 (1.37)	503 ± 8 (1.51)	574 ± 16 (1.72)	509 ± 17 (1.53)	−0.32	3.9	7.99	224.8

Values in parentheses are mutagenic index; Mi = induction factor; m = mutagenic potential; LSD = least significant difference.

The reversion of *Salmonella* strains with acidic and basic fraction of DCM extracts are presented in Table 5, Table 6. DCM extract of basic fraction showed highest response of 12.5 in absence of S9 and 12.72 in presence of S9 fraction in terms of mutagenic index; 2.44 without S9 fraction whereas 2.46 along with S9 fraction in terms of induction factor; and 6.3 with and without S9 fraction in terms of mutagenic potential in strain TA98 (Table 5). TA98 strain displayed highest response in the mutagenic index (11.95 with and 11.97 without S9), induction factor (2.38 with and 2.39 without S9 fraction) and mutagenic potential (6.7 with and 6.8 without S9 fraction) while treated with DCM extract of acidic fraction (Table 6). On the basis of mutagenic index and induction factor, the order of responsiveness in the presence as well as absence of S9 fraction for DCM (acidic and basic) fractions was found to be TA98>TA97a>TA100>TA102>TA104. The responsiveness order of tester strains of mutagenic potential/slope was different with mutagenic index and induction factor in DCM extracts (acidic and basic fractions). TA98 showed maximum number of revertants followed by TA100, TA97a, TA104 and TA102.

**Table 5:** Reversion of *Salmonella* tester strains in the presence of basic fraction of dichloromethane extracted wastewater.

Wastewater extract (μL/plate)											F value
Strain	S9	Control	2.5	5	10	20	40	Mi	m	LSD (*p*≤0.05)	
TA97a	–	88 ± 13	200 ± 20 (2.26)	267 ± 18 (3.02)	320 ± 15 (3.61)	397 ± 16 (4.49)	302 ± 24 (3.42)	1.25	4.2	26.3	147.2
	+	98 ± 14	238 ± 12 (2.42)	297 ± 19 (3.03)	355 ± 18 (3.62)	442 ± 24 (4.51)	359 ± 13 (3.65)	1.25	5.1	21.3	213.1
TA98	–	35 ± 10	135 ± 17 (3.85)	238 ± 19 (6.80)	336 ± 21 (9.60)	438 ± 19 (12.5)	323 ± 19 (9.22)	2.44	6.3	28.7	184.6
	+	39 ± 8	151 ± 16 (3.88)	268 ± 13 (6.88)	376 ± 15 (9.65)	496 ± 18 (12.72)	373 ± 19 (9.56)	2.46	7.3	25.9	165.2
TA100	–	156 ± 15	257 ± 9 (1.65)	301 ± 13 (1.93)	386 ± 19 (2.47)	463 ± 20 (2.97)	360 ± 20 (2.31)	0.67	4.3	31.4	129.8
	+	164 ± 10	275 ± 17 (1.68)	322 ± 21 (1.96)	411 ± 13 (2.51)	489 ± 25 (2.99)	396 ± 10 (2.42)	0.68	4.8	18.6	204.3
TA102	–	235 ± 12	323 ± 15 (1.37)	402 ± 11 (1.71)	480 ± 17 (2.04)	536 ± 15 (2.27)	451 ± 26 (1.91)	0.24	4.4	27.6	178.1
	+	247 ± 12	351 ± 20 (1.41)	431 ± 16 (1.74)	507 ± 17 (2.05)	573 ± 8 (2.31)	475 ± 23 (1.92)	0.27	4.6	19.6	149.8
TA104	–	305 ± 12	366 ± 14 (1.19)	428 ± 20 (1.40)	493 ± 19 (1.61)	555 ± 17 (1.81)	470 ± 21 (1.54)	−0.19	3.6	14.5	167.0
	+	314 ± 19	381 ± 13 (1.21)	452 ± 17 (1.44)	526 ± 20 (1.67)	579 ± 25 (1.84)	496 ± 14 (1.57)	−0.16	3.9	14.6	200.5

Values in parentheses are mutagenic index; Mi = induction factor; m = mutagenic potential; LSD = least significant difference.

**Table 6:** Reversion of *Salmonella* tester strains in the presence of acidic fraction of dichloromethane extracted wastewater.

Wastewater extract (μL/plate)											F value
Strain	S9	Control	2.5	5	10	20	40	Mi	m	LSD (*p*≤0.05)	
TA97a	–	84 ± 13	202 ± 14 (2.40)	278 ± 18 (3.31)	340 ± 20 (4.04)	422 ± 14 (5.02)	304 ± 14 (3.62)	1.39	3.5	12.7	168.7
	+	90 ± 8	226 ± 19 (2.51)	301 ± 23 (3.34)	366 ± 16 (4.06)	453 ± 17 (5.03)	344 ± 17 (4.81)	1.40	4.9	13.9	163.1
TA98	–	35 ± 8	119 ± 12 (3.42)	231 ± 10 (6.66)	321 ± 15 (9.26)	414 ± 14 (11.95)	335 ± 19 (9.67)	2.38	6.7	11.1	331.2
	+	41 ± 10	145 ± 12 (3.55)	280 ± 15 (6.87)	379 ± 22 (9.31)	487 ± 15 (11.97)	394 ± 15 (9.68)	2.39	6.8	12.6	353.9
TA100	–	135 ± 12	219 ± 13 (1.62)	305 ± 15 (2.25)	417 ± 19 (3.09)	519 ± 16 (3.84)	391 ± 16 (2.90)	1.04	5.9	12.6	247.2
	+	143 ± 11	254 ± 21 (1.77)	337 ± 18 (2.35)	446 ± 22 (3.12)	557 ± 21 (3.89)	416 ± 23 (2.91)	1.06	6.0	16.1	165.8
TA102	–	227 ± 10	333 ± 8 (1.46)	454 ± 22 (2.00)	514 ± 18 (2.26)	561 ± 12 (2.47)	444 ± 14 (1.94)	0.38	4.0	12.07	206.7
	+	241 ± 10	355 ± 19 (1.47)	487 ± 11 (2.02)	547 ± 1 (2.27)	597 ± 23 (2.48)	478 ± 21 (1.98)	0.39	4.3	14.5	163.02
TA104	–	275 ± 7	356 ± 18 (1.29)	404 ± 18 (1.47)	481 ± 14 (1.75)	553 ± 14 (2.0)	459 ± 21 (1.67)	0.01	4.0	13.02	113.8
	+	289 ± 12	382 ± 15 (1.32)	432 ± 19 (1.49)	509 ± 17 (1.76)	582 ± 17 (2.01)	498 ± 22 (1.72)	0.01	4.4	14.6	100.9

Values in parentheses are mutagenic index; Mi = induction factor; m = mutagenic potential; LSD = least significant difference.

The XAD concentrated wastewater sample was observed most mutagenic compared to other extracts as evident from mutagenic index, mutagenic potential and induction factor, the values were observed at the dose of 20 μL/plate. XAD extracted sample exhibited maximum toxicity followed by Hexane and DCM extracts respectively. All the values were pointedly higher with respect to control in all strains and signifying strong mutagenicity.

### 3.4 *Allium cepa* chromosomal aberration assay

**Fig. 1:**
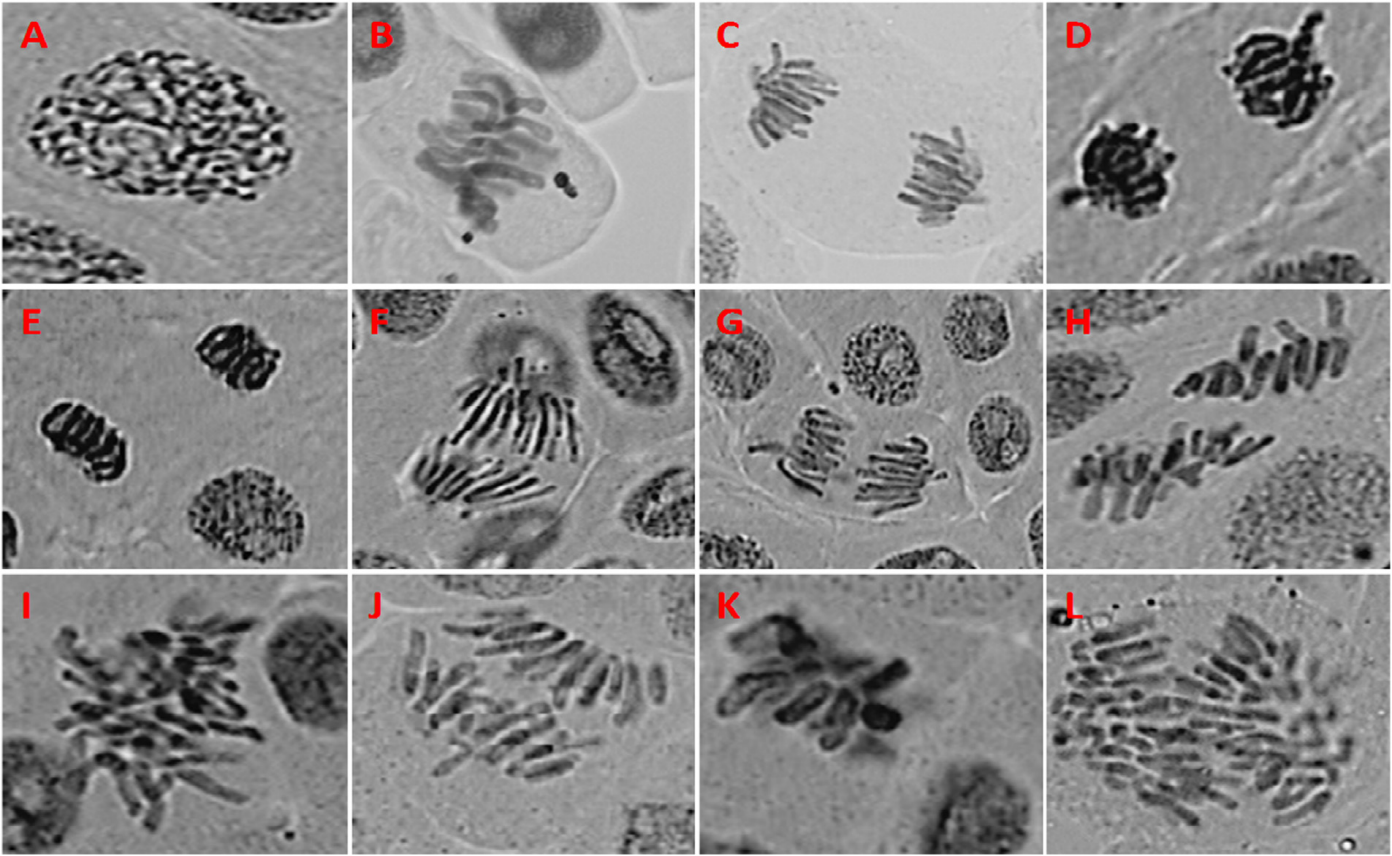
Normal phases and different types of chromosomal aberrations induced by the wastewater in *Allium cepa* root-tips A) Normal prophase, B) Normal metaphase, C) Normal anaphase, D) Normal telophase, E) Stickiness in telophase, F) Multipolar anaphase, G) Vagrant chromosome, H) Anaphase with chromosome break, I) Disturbed metaphase, J) Laggard chromosome, K) C-mitosis, L) Anaphase with chromosome break.

The genotoxic effects of wastewater on the MI and the incidence of mitotic phases of *A. cepa* on root meristematic cells are shown in Table 7. The value of MI was significantly decreased as the concentration of wastewater increased (26.7% at 5% and 11.03% at 100% wastewater concentrations). Highest MI (30.3%) was found in negative control (distilled water) while the cells treated with MMS shows the lowest MI (8.23%). Additionally, it was also observed that occurrence of mitotic phase was affected by the treatment, as the prophase cells percentage increased and metaphase cells decreased progressively with increase in wastewater concentrations upto 100%, while no uniform pattern was observed in anaphase-telophase stage. Moreover, the meristematic cells of root by treating with wastewater also revealed distinct forms of chromosomal aberrations such as Stickiness in telophase, Multipolar anaphase, Vagrant chromosome, Anaphase with chromosome break, Disturbed metaphase, Laggard chromosome, C-mitosis, Metaphase with chromosome break (Table 7; Fig. 1). The aberrant cells percentage was increased on increasing the wastewater concentration. Cells treated with MMS (positive control) showed highest number of aberrations however distilled water treated cells show very rare aberrations. The statistical studies of the sample showed that MI and percent abnormal cells triggered by treating along with wastewater were significant (P < 0.05) and relatively distinct from the positive and negative control samples by DMRT.

**Table 7:** Effect of different concentrations of wastewater on mitotic index and mitotic phase of *Allium cepa* root meristematic cells.

Samples	Concentration (% v/v)	Mitotic Phases (%)			Mitotic index (%±SD)
		Prophase	Metaphase	Anaphase-Telophase	
Wastewater	5	46.97	22.83	30.2	26.70 ± 1.0^b^
	10	46.52	23.08	30.4	19.17 ± 3.7^ab^
	25	54.54	16.96	28.5	16.67 ± 0.6^ab^
	50	55.2	19.8	25.0	13.46 ± 1.4^ab^
	100	58.85	26.2	14.95	11.03 ± 1.7^a^
Positive control		63.63	22.72	13.65	8.23 ± 1.6^a^
Negative control		49.5	31.75	18.75	30.3 ± 2.4^c^

Means with the same letters do not significantly differ at 0.05 level (Duncan multiple range test); **±**: Standard deviation.

### 3.5 *In vitro* toxicity assessment of wastewater to *V. Rradiata*

**Fig. 2:**
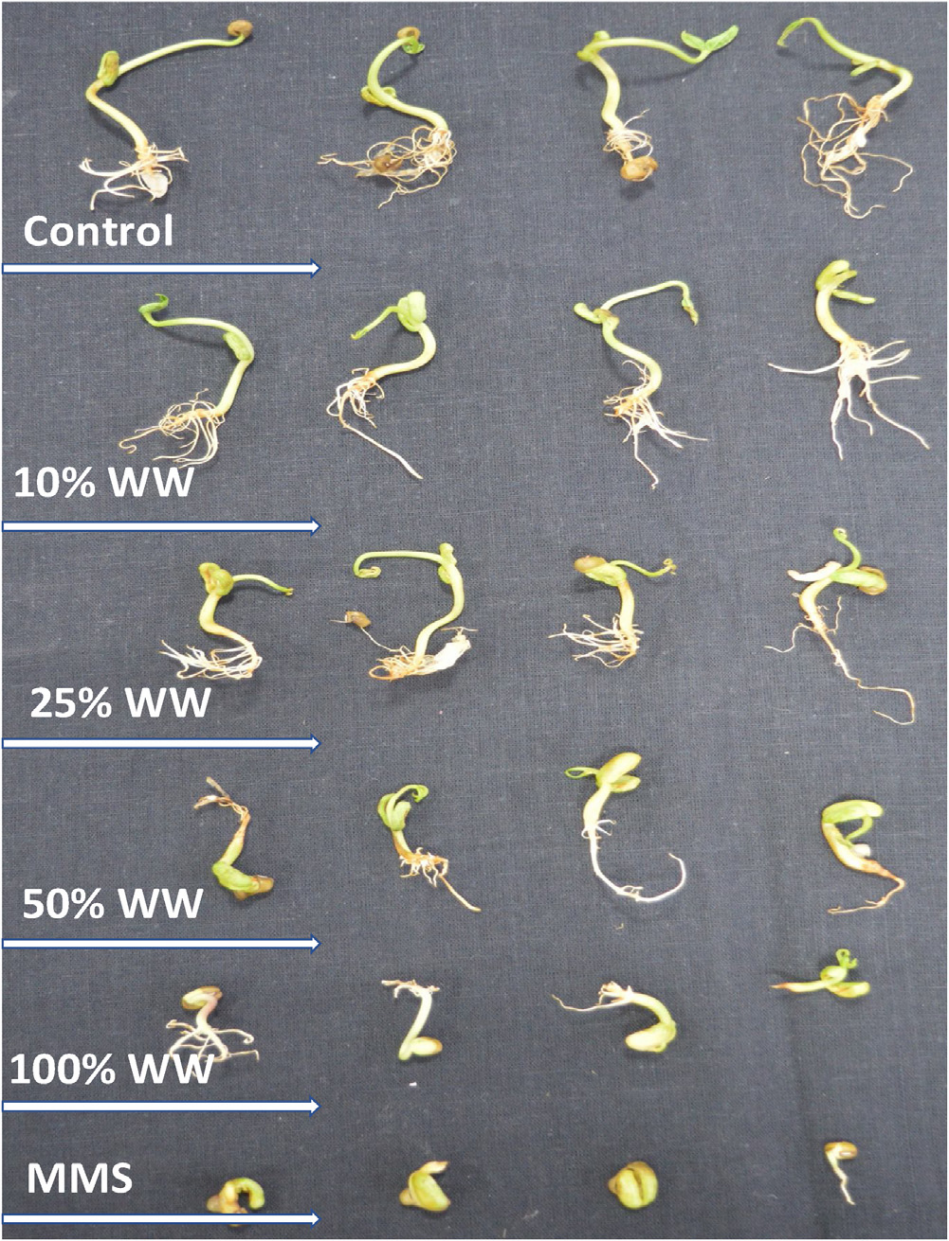
Dose-dependent reduction in the radicle and plumule length of mung bean seeds germinated on 0.7% agar plates amended with 10%, 25%, 50% and 100% wastewater concentrations.

**Fig. 3:**
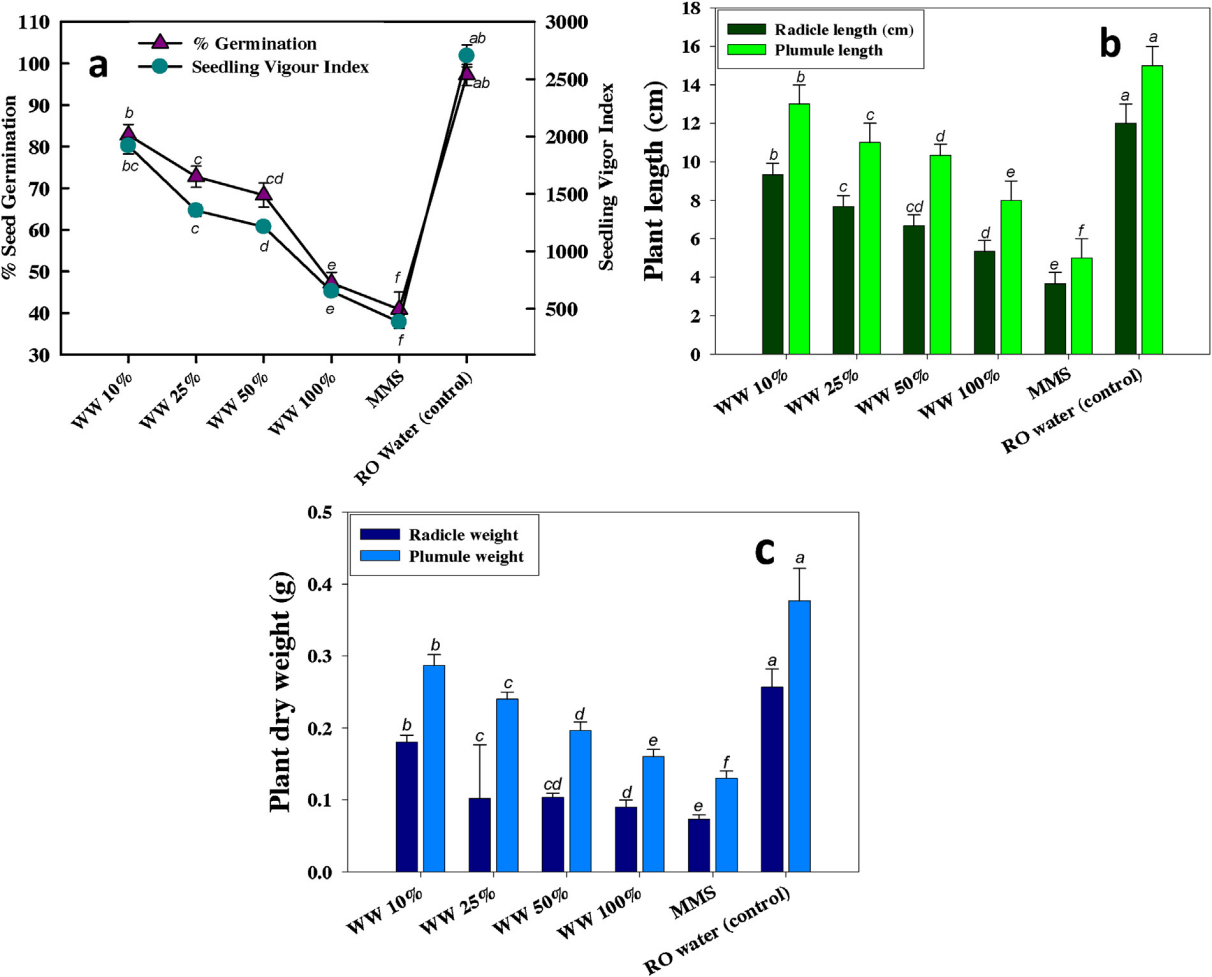
Plant parameters of *Vigna radiata* (mungbean) seeds germinated on soft agar plates treated with different (10, 25, 50 and 100%) concentrations of wastewater; % germination and seedling vigor index (SVI) (a), radicle and plumule length in (cm) (b) and dry biomass (c). Each value is a mean of five independent replicates (n = 5) where each replicate constituted five seeds/plates. Mean values followed by different letters are significantly different at *p≤*0.05 according to Tukey’s-b test. Vertical bars represent means ± SD (n = 5).

**Fig. 4:**
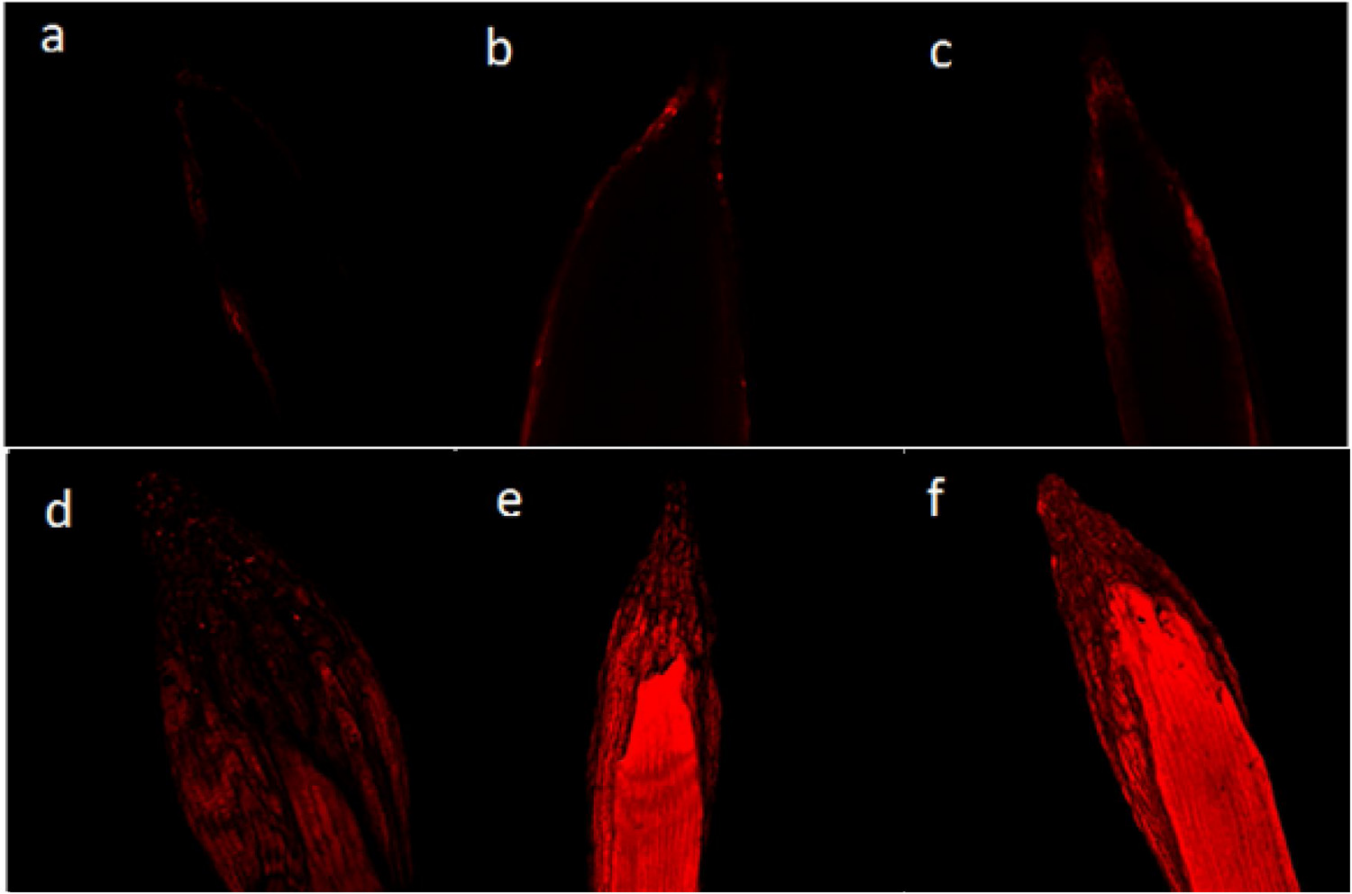
Confocal laser scanning microscopic (CLSM) images of *Vigna radiata* roots stained with propidium iodide (PI) and treated with different concentrations of wastewater; control (a), treated with 10% (b), 25% (c) 50% (d) 100% (e) and MMS (positive control) (e). As the treatments of wastewater increased, the uptake of dye (PI) also increased.

We also assess the toxic behaviour of wastewater on another plant i.e. *V. radiata* (mung bean), to confirm the toxicity. Under untreated condition, percent germination of seed, seedling vigor index (SVI), radicle length (RL), plumule length (PL), dry biomass of radicle (DBR) and dry biomass of plumule (DBP) was found to be 97%, 2610 SVI, 12, 15 cm, 0.25 and 0.37 gm, respectively (Fig. 3). However, all these plant parameters were reduced as the concentration of wastewater increased. Percent germination, SVI, RL, PL, DBR and DBP were significantly decreased by 52%, 76%, 56%, 47%, 64% and 57%, respectively (Fig. 3), when grown on soft agar plates amended with highest concentration of wastewater (Fig. 2). The damage in the cells of root tips due to wastewater were observed and clearly visible under fluorescent microscope with red fluorescence produced by propidium iodide. The intensity of fluorescence continuously enhanced while increasing wastewater concentration (Fig. 4).

### 3.6 Plasmid nicking assay

**Fig. 5:**
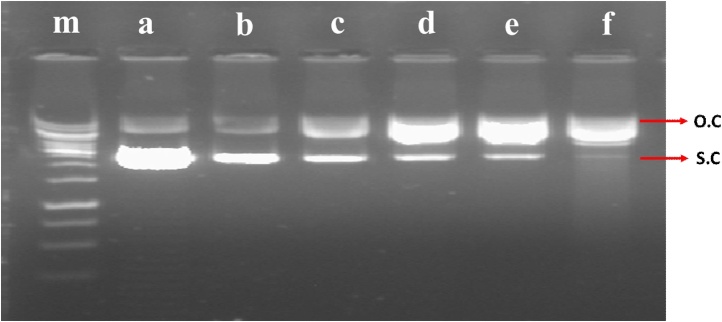
Plasmid-nicking assay conducted on the wastewater-samples. Lane m: 1 kb ladder Lane a pBR322 DNA alone. Lane b-e: pBR322 DNA + 5 μl, 10 μl, 15 μl, 20 μl of wastewater respectively. Lane f: pBR322 DNA + MMS.

Fig. 5 presents the DNA band profiles obtained after the pBR322 plasmid nicking assay with a series of wastewater concentration. The test volume of 5 to 20 μl of the wastewater sample in a 25 μl reaction mixture resulted in the conversion of pBR322 DNA from supercoiled form into the open circular (Fig. 5; lane a). The intensity of open circular form of plasmid was increased on increasing the wastewater concentration and the band intensity of supercoiled form was decreased to complete loss of supercoiled form (Fig.5; lane b–e). The maximum conversion of supercoiled to open circular was observed in pBR322 plasmid treated with MMS (Fig. 5; lane f).

## 4 Discussion

Ghaziabad district is one of the key industrial hubs in Northern region of India, the location of wastewater sampling site is 28°44′N and 77°17′E, and a number of industries including the pesticide industries, that produce vast quantity of wastewater that are steadily discharged into the river. Physico-chemical parameters employed to observe quality of water were TDS, carbonate, bicarbonate, chloride and sulphate were above the permissible limits as defined by USEPA (Table 1). Increase in the TDS is also indicating the pollution level of water that marks the self-purification process of the wastewater and also harmful for aquatic animals due to osmotic stress [34]. In addition to physico-chemical analysis, toxicity evaluation of wastewater is of applied significance as it would support in expecting the collective effects of diverse compounds into the water. Various heavy metals existing in the wastewater demonstrated deleterious influence on the environment and health of humans [8]. In our previous studies, we have reported the existence of high concentrations of several metals in wastewater and contaminated soil [28,35]. The detection of specific organic substances along with mutagenic activity in untreated wastewater or even in effluents of industries is quite problematic, because only a very limited compounds are found in the detectable limit.

Environmental pollution of wastewaters by residues of pesticide is of great concern. Insecticides are a group of organic compounds that shows broad range of toxic effects and ultimately cause a potential threat to the environment [36,37]. EI-Gawad [38] detected several pesticides of organochlorine group such as Alpha-BHC, Gamma-BHC, Aldrin, Heptachlor, Heptachlor epoxide, in water samples at high concentrations. Toxicity of Cr and Ni are reported in lipid peroxidation, generation of reactive oxygen species (ROS), oxidative stress and DNA damage [39].

High level of organochlorine and organophosphate pesticides (Table 2) are reported in past [11,36]. Bedi et al [40] reported persistent organic pollutants containing lindane, DDE, DDD, endosulfan sulfate as well as polychlorinated biphenyl in the fish sample. In Nigeria, the surface water of fifteen different sites of two river were evaluated for the quantification of twenty organochlorine pesticides [41]. They also detected the organochlorine pesticides in brackish fish (*Drepane africana* and *Mochokus niloticus*) samples of the Niger River with concentration range of 2237–6368 μg/kg of fresh weight.

The present study showed the genotoxic, cytotoxic and mutagenic potential of the wastewater. The combined effect of cytotoxicity and genotoxicity including bacterial (prokaryotic) and plant (eukaryotic) entity, were performed to obtain a thorough impact of wastewater on the environment. These analyses are significant for the assessment of harmful waste and threat calculation correlated with contaminants [[42], [43], [44]]. A huge number of mutagens were extracted in different organic solvents (dichloromethane, n-hexane, ethyl acetate, acetone, acetonitrile etc.) and were identified including aromatic amines, polycyclic aromatic hydrocarbons, polychlorinated compounds [21]. The industrial effluents comprising diverse range of chemicals that have been found to be genotoxic and responsible for various stages of DNA damages in the organisms of aquatic system [17]. Due to complex substances present in wastewater samples, a single test cannot assess all the mode of toxicity in the samples that are mixtures of contamination [45,46]

In the Ames *Salmonella*/microsome assay the tester strains contain a certain alteration in ‘histidine operon’ (i.e., TA97a / TA98 frameshift mutations, strain TA100 base pair substitution / missense mutations and TA102 / TA104 transitions / transversions) and hence distinguish a particular form of mutagen [20,47]. Rehana et al [48] also used five different Ames *Salmonella* bacterial strains to test genotoxicity of Ganges river water at different sites and observed that TA98 and TA100 displayed high mutagenicity with and without S9 fraction. Numerous workers observed that in XAD concentrated extracts, TA98 strain was more responsive compare to TA100 in both with and without S9 fraction, moreover extracted concentration of XAD was also more mutagenic than the samples of liquid-liquid extraction, as reported by several workers [8,49]. Wastewater discharge from industrial area of Lucknow (pesticide industry) is used for irrigation purposes and the soil irrigated with wastewater showed strong mutagenic activity in comparison to soil irrigated with ground water [50].

The life cycle of *A*. *cepa* root meristematic cells is short (20 h) and contains smaller number of chromosomes (2n = 16) compare to other plants. That’s why it is preferable eukaryotic plant system for the evaluation of damages in chromosomes [30]. Cytotoxicity and genotoxicity of wastewater were assessed by detecting different cytological parameters for example mitotic index and aberrations in chromosomes including breaks in chromosomes, laggard chromosome, C-mitosis, anaphase bridges and stickiness. The two important parameters were used i.e. reduction in mitotic index and increase in chromosomal aberration for assessing genotoxicity and cytotoxicity of numerous compounds present in the test samples [51]. Many earlier reports have confirmed that *A. cepa* and mammalian test systems has good correlation [51,52]. In this study we observed a significant reduction in mitotic index as concentration of wastewater increased in comparison of the control (Table 7), this is due to high level of trace elements in single or in combination of other metals have inhibitory effect on cell division [[53], [54], [55]]. The wastewater is responsible for decline in mitotic index in roots of *A. cepa* due to toxicity of pesticides, heavy metals and many other pollutants, ultimately cell death occurred [[56], [57], [58]].

The chromosomal aberrations are direct indicative of DNA damage that could not be easily repaired [59]. In the present study, several types of aberrations in the chromosomes were observed i.e. C-mitosis, disturbed metaphase and vagrant chromosomes being most distinguished (Table 8; Fig. 1). C-mitosis is occurred due to the spindle disturbance in mitotic phase [60]. The vagrant chromosomes observed because of failure of chromosomal separations in the stage of metaphase [61] and risk of aneuploidy increased [51]. Thus, the occurrence of several type of aberrations in chromosomes (C-mitosis, disturbed metaphase, stickiness, vagrant chromosomes etc.) in the meristematic cells of *A. cepa* root could be attributed to collective effect of clastogenic as well as aneugenic actions of several compounds in the wastewater [62].

**Table 8:** Chromosomal aberrations in the root meristematic cells of *Allium cepa* exposed to different concentrations of wastewater for 72 h.

Sample	Concentration (% v/v)	Types of aberrations								Total aberrant cells (%±SD)
		VC	CM	LC	MA	DM	SC	AB	DAT	
Wastewater	5	2	1	2	–	–	1	1	–	5.53 ± 0.72^e^
	10	2	2	3	–	2	2	–	1	10.10 ± 1.850^d^
	25	4	6	4	–	3	3	5	3	20.15 ± 1.56^c^
	50	5	2	8	7	6	1	5	4	28.98 ± 4.77^c^
	100	10	7	4	4	8	1	10	7	34.26 ± 2.98^b^
Positive control		13	12	10	3	5	12	11	14	40.97 ± 2.66^a^
Negative control		3	–	1	–	2	–	1	2	5.82 ± 0.32^e^

CM: C-mitosis, AB: anaphase bridge, LC: laggard chromosome, BN: binucleated cell, S: stickiness, DM: disturbed metaphase DAT: disturbed anaphase-telophase, VC: vagrant chromosome; MA: multipolar anaphase; Means with the same letters do not significantly differ at 0.05 level (Duncan multiple range test); **±**: Standard deviation.

The cell membrane is one of the important, selectively permeable organelles that controls the exchange of ions and molecules and permit the cells to communicate with the neighbouring environment. CLSM has proved to be the most delicate and reliable technique for obtaining a 3D image of basic tissue. Seed germination is dynamic phenomenon during the life cycle of plants therefore, SG and SVI is considered as the most significant physical characteristics of seeds that are used for cultivation. In this context, delayed germination following the application of wastewater has been associated with disturbed germinative metabolism which is a complex process. The toxic impact of pesticide on the germination efficiency of *Dimorphandra wilsonii*, belongs to Fabaceae family has been reported [63]. Similarly, the reduced length of radicle and plumule in germinated seeds of pea due to the toxic influence of another environmental stressor molecule (pesticide) has recently been reported [33].

The breakage in the length of DNA strand is a significant aspect to evaluate mutagenic impact of several chemical substances on integrity of DNA. The damage or break in DNA is caused by an exogenous agent or it may be formed in the repair processes of DNA, or physiologic responses into the cell [64]. In this study, the plasmid nicking assay also revealed genotoxic and mutagenic potential of wastewater. (Fig. 5).

## 5 Conclusion

The physico-chemical, GC and AAS analysis of wastewater revealed numerous genotoxic substances in the form of organic and inorganic pollutants which are directly or indirectly harmful for ecosystem and human health. A set of bacterial and plant-based tests demonstrated that the wastewater showed mutagenicity and genotoxicity by reverting the *Salmonella* tester strains (TA97a, TA98, TA100, TA102 and TA104). Strain TA98 showed highest response in the terms of induction factor, mutagenic index as well as mutagenic potential. The industrial wastewater also comprised of phytotoxic and cytotoxic substances, that’s why decrease in mitotic index occurred and caused different forms of chromosomal abnormalities in meristematic cells of *A. cepa*. The effect of wastewater on *V. radiata* showed decreased percent seed germination, reduced length (radicle and plumule) and uptake of propidium iodide as observed under CLSM. Furthermore, the wastewater also induced damage in the naked DNA in plasmid nicking assay. As evident by an array of cytotoxic and genotoxic assays, it is recommended that the effluents from the industries should be treated appropriately to minimize the presence of the genotoxic and cytotoxic compounds before entering into the river system.

## Declaration of Competing Interest

The authors declare that they have no conflict of interest.
